# Decoding Urinary Stones: Compositional Insights and Recurrence Patterns from a Tertiary Care Hospital in Eastern India

**DOI:** 10.7759/cureus.70136

**Published:** 2024-09-24

**Authors:** Prem Kumar, Shamim Ahmad, Pranjal Prem, Himangshu Mazumdar, Kumari Asha Kiran, Smita Singh

**Affiliations:** 1 Urology, Ranchi Urology Centre, Ranchi, IND; 2 Pathology and Laboratory Medicine, Dr Lal PathLabs, New Delhi, IND; 3 Preventive Medicine, Rajendra institute of Medical Sciences, Ranchi, IND; 4 Obstetrics and Gynaecology, Ranchi Urology Centre, Ranchi, IND

**Keywords:** stone analysis, stone composition, stone recurrence, urinary stone, urolithiasis in eastern india

## Abstract

Background and objective

Urinary stones are a prevalent medical condition entailing significant health burdens and substantial financial ramifications. Its global prevalence is expected to rise notably, particularly in low-middle-income nations. Understanding the spectrum of diverse urinary stone types is crucial for effective management and prevention. This study aims to elucidate the demographic profiles, clinical types, and recurrence of urinary stone cases at a tertiary care hospital in Eastern India.

Methods and materials

The clinical data from the electronic medical record of 1,231 patients with urolithiasis who underwent surgery in a tertiary care center in eastern India from January 2015 to November 2022 were retrospectively analyzed. Patient data, including demographic information, clinical records, dietary habits, water intake, and stone recurrence history, were collected. A thorough statistical analysis was conducted to elucidate the associations between patient characteristics, urinary stone composition, and recurrence.

Results

Among the 1,231 participants, the majority of cases (343 (27.9%)) were in the 31-40 age group, with a higher prevalence in males (876 (71.2%)) than in females (355 (28.8%)). Flank pain or abdominal pain (593 (48.1%)) was the primary complaint, followed by nausea/vomiting (227 (18.5%)). Most stones (922 (74.9%)) were located in the kidney, and calcium oxalate was the predominant stone component (773 (62.8%)). The highest stone recurrence rates were in patients aged between 21-30 years (21 (36.8%)) and 31-40 years (16 (28.0%)). A low non-vegetarian diet and adequate hydration may reduce the likelihood of stone recurrences.

Conclusion

This study provides insights into the compositional analysis of urinary stones in the eastern Indian population, addressing the causes of their recurrence and management strategies, which are crucial for prevention and effective management. The findings indicate that the early middle-aged group exhibited the highest incidence of urinary stones. We also observed that strict adherence to a low animal protein, along with proper hydration and lifestyle changes, significantly reduced urinary stone recurrence.

## Introduction

Urinary stones are hard, crystalline mineral deposits that form within the urinary tract, composed primarily of substances such as calcium oxalate, uric acid, struvite, and cystine. The complexities associated with urinary stones extend beyond their formation. They often lead to severe complications such as urinary obstruction, recurrent infections, and chronic kidney disease [1].

Over the past decade, there has been a notable increase in the global incidence of urinary stone disease [2], attributed to lifestyle changes, environmental factors, and the widespread use of medications, including self-medication [3]. The multifactorial nature of urinary stone disease makes it challenging to identify a single definitive etiological factor. The condition is further complicated by its high recurrence rate [4]. The incidence of urolithiasis varies across different countries, regions, races, sexes, and age groups [5].

Compositional analysis of urinary stones is crucial for understanding their pathogenesis and guiding effective management strategies. The crystalline composition of a stone reflects the urine chemistry and abnormalities during the process of stone development. Accurate knowledge of the stone composition is critical in elucidating the underlying etiology of the patient’s clinical disorder that precipitated the stone disease [1].

The management of urinary stones has evolved significantly with the advent of innovative treatment modalities such as extracorporeal shock wave lithotripsy (ESWL), percutaneous nephrolithotomy (PCNL), flexible or semi-rigid ureteroscopy, and transurethral/percutaneous cystolithotripsy. These techniques have revolutionized urinary stone management by improving efficacy and reducing surgical complexity [6-9]. Despite these advances, addressing the root causes of urinary stone disease remains crucial, as current procedures primarily focus on stone removal rather than preventing recurrence [10].

According to the European Association of Urology (EAU) guidelines, it is recommended that every patient with urinary stones should undergo stone analysis, as knowing the stone composition is crucial for patient evaluation and treatment planning [11, 12]. Therefore, all urinary stones should be analyzed to guide appropriate management strategies.

Our study addresses the gaps in existing findings related to urinary stone prevalence, composition, and recurrence in eastern India. By decoding these insights, we aim to tailor effective management and preventive strategies for urinary stones.

## Materials and methods

In this retrospective, hospital-based observational study, we meticulously analyzed electronic medical records (EMR) from 1,231 patients who underwent urinary stone surgery at Ranchi Urology Centre, Ranchi, a tertiary care center in eastern India between January 2015 and November 2022. Written informed consent was obtained from all patients to use their data for research purposes at the time of surgery. Ethical approval from Ranchi Urology Centre's Institutional Ethics Committee was obtained (reference number: RUC IEC 04/23) for retrospective analysis of this patient cohort. We collected comprehensive patient data, including demographic information, clinical records, dietary habits, water intake, and stone recurrence history. Subsequently, a rigorous statistical analysis was conducted to elucidate the coalition between patient characteristics, urinary stone composition, and the likelihood of recurrence.

Study population

The study population consisted of patients admitted to Ranchi Urology Centre for stone removal. These patients underwent surgical intervention for both primary and recurrent urolithiasis.

To investigate potential variations in stone composition across different age groups, patient data were stratified into distinct cohorts, with age ranges spanning from one to 10 years to 81-90 years. Patient-specific data, including clinical details, demographic information, stone localization, recurrence history, and stone composition reports, were meticulously documented. Patients were followed up during scheduled routine OPD visits. Those who were lost to follow-up were contacted telephonically for recurrence analysis.

Process of collecting study data

After surgical retrieval, the stones were sent for detailed stone analysis. The collected urinary stones underwent thorough cleansing using distilled water, ensuring the removal of any residual blood or tissue. Subsequently, the stones were carefully dried using filter paper. This scrupulous preparation preceded the compositional analysis through a Fourier transform infrared spectroscope (FTIR). All the results obtained during this analysis were transcribed into the EMR, serving as essential data for our subsequent retrospective study.

In this study, data were collected from patients across all age groups who had undergone stone surgery, where specimens of stone or stone fragment were available, and for whom a stone analysis report was available in the EMR. There were no exclusion criteria applied.

Statistical analysis

The data were initially entered into a Microsoft Excel spreadsheet (Microsoft Corp., Redmond, WA) and then imported into the statistical analysis software IBM SPSS Statistics software for Windows, version 16 (IBM Corp., Armonk, NY) for data analysis. Descriptive analysis included measures such as mean or median with standard deviation (SD), proportions, and percentages. Additionally, a chi-square test was conducted to assess the significance of the data, with a p-value <0.05 considered statistically significant.

## Results

In the course of our study, the investigation into urinary stone disease revealed distinct gender-related trends. Among the 1,231 cases studied, a significant difference in the incidence of calculi was observed when comparing the male-female ratio. The majority of calculi (876 (71.2%)), were observed in male patients, while 355 (28.8%) cases were in female patients. Patient ages spanned a wide range from one to 90 years, with a calculated mean age of 39.13 years. Male patients exhibited a slightly lower mean age (38.59 years) compared to female patients (40.47 years).

Urinary stones were most prevalent among younger patients, with 343 (27.9%) occurring in the 31-40 age group and 286 (23.2%) in the 21-30 age group. In contrast, the elderly population had much lower rates, with only four (0.3%) cases in the 81-90 age group and nine (0.7%) cases in the 71-80 age group.

The majority of urinary stones in this study were of renal origin, comprising 922 (74.9%) cases. Among these, 652 (53%) cases occurred in male patients, while 270 (21.9%) cases occurred in female patients. Ureteric stones constituted 217 (17.6%) cases, with 147 (11.9%) cases in males and 70 (5.7%) cases in females. Stones originating from the bladder accounted for 92 (7.5%) cases, with 75 (6.1%) cases observed in males and 17 (1.4%) cases in females.

The predominant symptoms reported by most patients in the study were flank pain or abdominal pain in 593 (48.1%) cases, followed by nausea/vomiting in 227 (18.5%) cases, as shown in Table 1.

**Table 1:** Urinary stone analysis according to patient characteristics Correlation of urinary stone composition with patient demographics and clinical profiles

Parameters		Urinary stones (calculi)		
		Overall n(%)	Male n(%)	Female n(%)
		1231	876 (71.2) *	355 (28.8) *
Mean age (Mean±SD)		39.13±14.5	38.59±14.7	40.47±13.8
Age range (years)	1-10	35 (2.8)	25 (2.01)	10 (0.81)
	11-20	51 (4.1)	38 (3.08)	13 (1.05)
	21-30	286 (23.2)	210 (17.1)	76 (6.1)
	31-40	343 (27.9)	251 (20.4)	92 (7.4)
	41-50	244 (19.8)	165 (13.4)	79 (6.4)
	51-60	171 (13.9)	110 (8.9)	61 (5.0)
	61-70	88 (7.1)	65 (5.3)	23 (1.9)
	71-80	09 (0.7)	08 (0.6)	1 (0.1)
	81-90	04 (0.3)	04 (0.3)	00 (0)
Stone location	Kidney	922 (74.9)	652 (53)	270 (21.9)
	Bladder	92 (7.5)	75 (6.1)	17 (1.4)
	Ureter	217 (17.6)	147 (11.9)	70 (5.7)
Chief complaints	Flank pain/abdominal pain	593 (48.1)	421 (34.2)	172 (13.9)
	Nausea/vomiting	227 (18.4)	151 (12.3)	76 (6.2)
	Hematuria	167 (13.6)	110 (8.9)	57 (4.7)
	Lower urinary tract symptoms (LUTS)	135 (11.0)	41 (3.4)	94 (7.7)
	Fever with chills	105 (8.5)	73 (5.9)	32 (2.6)
* The p-value for the difference in occurrence between males and females is significant, determined by the non-parametric binomial test.				

In the analyzed stones, single-component pure stones constituted 71 (5.77%) of the total, while two-component stones were the most prevalent, accounting for 1,065 (86.5%). Three-component stones comprised 95 (7.71%) of the overall composition.

Among single-component stones, ammonium hydrogen urate stones were the most common, representing 22 (1.79%) of the total. For two-component stones, the predominant composition was a combination of calcium oxalate monohydrate and calcium oxalate dihydrate, totaling 773 (62.8%). Among three-component stones, the most frequently observed combination was calcium oxalate monohydrate, calcium oxalate dihydrate, and carbonate apatite, representing 73 (5.93%) of the total, as shown in Table 2.

**Table 2:** Composition-based distribution of urinary stones (pure, two-component, and three-component) Distribution of urinary stones as pure, two-component, and multiple-component formations.

Stone composition		Number (n)	Percentage (%)
Pure stone			
Ammonium hydrogen urate		22	1.79
Cystine		17	1.38
Uric acid		15	1.21
Calcium oxalate (monohydrate)		11	0.89
Carbonate apatite		4	0.32
Struvite		1	0.08
Xanthine		1	0.08
Total		71	5.77
Two-component stone			
Calcium oxalate (MH)+ Calcium oxalate (DH)		773	62.8
Calcium oxalate (DH)+ Carbonate apatite		234	19
Calcium oxalate (MH)+ +Xanthine		15	1.21
Calcium oxalate (MH)+ Uric acid		12	0.97
Calcium oxalate (MH)+ Carbonate apatite		10	0.81
Calcium oxalate (DH)+ Cystine		10	0.81
Carbonate Apatite +Struvite		5	0.4
Calcium oxalate (MH) + Struvite		2	0.16
Calcium oxalate (MH)+ Cystine		1	0.08
Ammonium hydrogen urate + Uric acid		1	0.08
Calcium oxalate (DH) + Struvite		1	0.08
Calcium oxalate (MH) + Ammonium hydrogen urate		1	0.08
Total		1065	86.5
Three-component stone			
Calcium oxalate (MH) + Calcium oxalate (DH) + Carbonate apatite		73	5.93
Calcium oxalate (MH) + Carbonate apatite + Struvite		19	1.54
Ammonium hydrogen urate + Uric acid + Cystine		1	0.08
Calcium oxalate (DH)+ Struvite + Uric acid		1	0.08
Ammonium hydrogen urate + Calcium hydrogen phosphate + Magnesium hydrogen phosphate		1	0.08
Total		95	7.71

**Figure 1:**
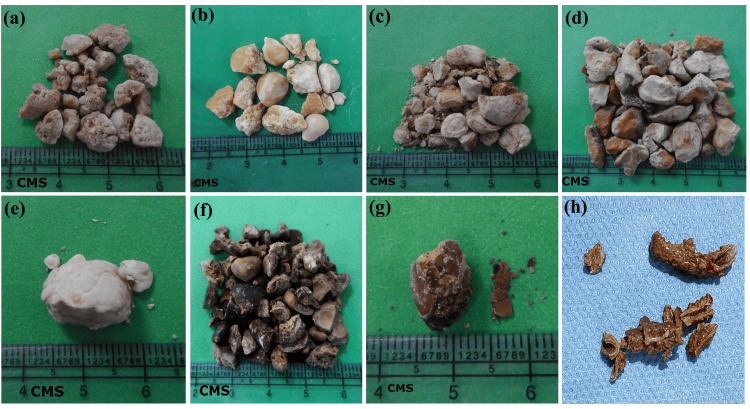
Postoperative urinary stones of different compositions (a) Calcium oxalate monohydrate with calcium oxalate dihydrate stone; (b) Cystine stone; (c) Struvite stone; (d) Uric acid stone; (e) Carbonate apatite stone; (f) Calcium oxalate monohydrate stone; (g) Ammonium hydrogen urate stone; (h) Stone over double J stent (pure carbonate apatite) CMS: centimeters

In the context of stone removal procedures, percutaneous nephrolithotomy (PCNL) was the most commonly used procedure in 905 (73.5%) cases. Following this, ureteroscopy was used in 238 (19.3%) cases, cystolithotomy/laser cystolithotomy in 81 (6.58%) cases, and pyelolithotomy in seven (0.6%) cases. Figure 1 showcases a range of postoperative stone images, illustrating the diverse types of stones encountered after surgical procedures.

In another context, the data analysis showed that two-component stones were the most common type of urinary stones across all age groups and locations. The highest number of two-component stones were found in the 31-40 year age group, followed by the 21-30 year age group.

Conversely, one-component and multiple-component stones were most frequently found in the 21-30-year age group, followed by the 31-40-year age group.

Kidney stones were the most prevalent among all urinary organs across all stone composition groups, with the highest number being two-component stones, comprising 821 (66.6%) cases.

There was a significant difference between age range and stone composition, as well as between the stone location and composition, across all age groups (Table 3).

**Table 3:** Age-dependent composition trends in urinary calculi Correlation between stone components and different age groups

Parameters		One-component stone n(%)	Two-component stone n(%)	Multiple-component stone n(%)	Ch-square test	
					Chi-square, df	p-value
Age range (years)	1-10	03 (0.24)	24 (1.94)	08 (0.64)	188.0, 16	P<0.0001
	11-20	07 (0.56)	32 (2.6)	12 (0.97)		
	21-30	20 (1.62)	246 (19.2)	20 (1.62)		
	31-40	17 (1.38)	310 (25.2)	16 (1.29)		
	41-50	11 (0.89)	223 (18.11)	10 (0.81)		
	51-60	06 (0.49)	157 (12.7)	08 (0.64)		
	61-70	07 (0.57)	72 (5.9)	09 (0.73)		
	71-80	00 (0)	00 (0)	09 (0.74)		
	81-90	00 (0)	01 (0.1)	03 (0.24)		
	Total	71 (5.77)	1065 (86.52)	95 (7.72)		
Stone location	Kidney	56 (4.54)	821 (66.6)	45 (3.65)	49.97, 4	P<0.0001
	Bladder	05 (0.40)	66 (5.36)	21 (1.7)		
	Ureter	10 (0.81)	178 (14.5)	29 (2.35)		
	Total	71 (5.77)	1065 (86.52)	95(7.72)		

**Figure 2:**
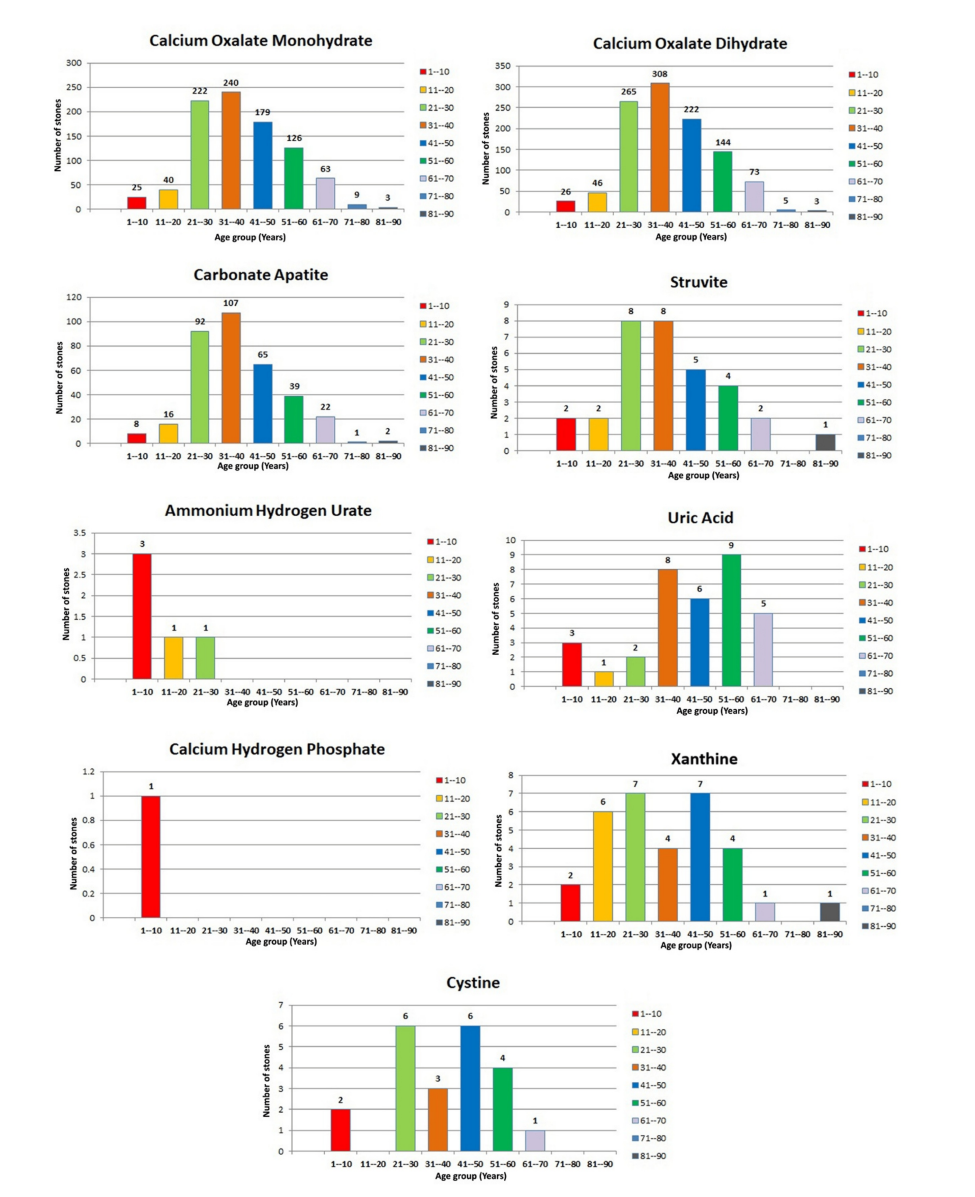
Graphical representation of urinary stone composition in different age groups

It is revealed that individuals aged 31-40 years exhibited the highest prevalence of calcium oxalate monohydrate stones, with 240 (19%) cases; calcium oxalate dihydrate stones, with 308 (25%) cases; and carbonate apatite stones, with 107 (8.7%) cases. This trend was closely followed by the 21-30 year age group. Struvite stones were equally prevalent in the 21-30 and 31-40 age groups, each accounting for eight (0.6%) cases. Ammonium hydrogen urate and calcium hydrogen phosphate stones were predominantly observed in the pediatric group aged one to 10 years. Moreover, xanthine stones were equally distributed between the 21-30 and 41-50 age groups, each comprising seven (0.56%) cases, as shown in Figure 2.

This underscores a notable age-dependent distribution of various urinary stone types in eastern India. Particularly, the prevalence observed among individuals aged 21 to 50 years points to dietary patterns and metabolic factors prevalent in this demographic. These insights emphasize the critical importance of early dietary interventions and metabolic assessments to mitigate the incidence of urinary stones across diverse age groups. Tailored dietary modifications aimed at reducing the consumption of stone-forming constituents hold promise for effectively managing this condition.

Urinary stone recurrence analysis

The study observed a cohort of 1,231 patients diagnosed with urinary stones, with a subset of 530 individuals undergoing follow-up evaluations. Within this subgroup, 57 (10.75%) patients experienced stone recurrence. The recurrence rate begins to increase in patients aged between 11 and 20 years, reaching its peak in the 21-30 age group, followed by high incidence numbers in the 31-40 age group. In patients aged between 41 and 50 years, the rate started to decline, and there were no recurrences observed in older patients within the 71-80 and 81-90 age groups.

The analysis showed no significant difference between stone recurrence and factors such as age, gender, diet, water intake, and anatomical location. However, of patients between the ages of 21 to 50 years, 47 (82.4%) reported the highest number of recurrence cases. Of the total recurrence cases, 38 (66.6%) patients preferred a frequent non-vegetarian diet, and 40 (70.1%) were those who had a daily water intake of less than or equal to 2 liters.

Gender-wise, 39 (68.4%) patients were male, and 33 (57.9%) patients with stones anatomically located in the kidney had the most recurrences, as shown in Table 4.

**Table 4:** Recurrence of urinary stones: predominance in young adults Analysis of urinary stone recurrence with reference to various factors. veg: vegetarian; non-veg: non-vegetarian; lit: liters

Parameters		Age range									Chi-square test		
		01-10	11-20	21-30	31-40	41-50	51-60	61-70	71-80	81-90			
											Chi-square, df	p-value	
Recurrence cases (n=57/530)		1 (1.7)	6 (10.5)	21 (36.8)	16 (28.0)	10 (17.5)	1 (1.7)	2 (3.5)	0 (0)	0 (0)			
Diet	Veg diet	0 (0)	2 (3.5)	6 (10.5)	4 (7.0)	5 (8.7)	0 (0)	2 (3.5)	0 (0)	0 (0)	6.946,6	0.3242	
	Non-veg diet	1 (1.7)	4 (7.0)	15 (26.3)	12 (21.0)	5 (8.7)	1 (1.7)	0 (0)	0 (0)	0 (0)			
Water intake	≥ 4 lit water/day	0 (0)	1 (1.7)	5 (8.7)	7 (12.2)	3 (5.2)	0 (0)	1 (1.7)	0 (0)	0 (0)	3.581,6	0.7332	
	≤ 2 lit water/day	1 (1.7)	5 (8.7)	16 (28.0)	9 (15.7)	7 (12.2)	1 (1.7)	1 (1.7)	0 (0)	0 (0)			
Stone location	Kidney	0 (0)	3 (5.2)	15 (26.3)	9 (15.7)	5 (8.7)	1 (1.7)	1 (1.7)	0 (0)	0(0)	14.84,12	0.2505	
	Bladder	1 (1.7)	3 (5.2)	2 (3.5)	5 (8.7)	1 (1.7)	0 (0)	1 (1.7)	0 (0)	0(0)			
	Ureter	0 (0)	0 (0)	4 (7.0)	2 (3.5)	4 (7.0)	0 (0)	0 (0)	0 (0)	0(0)			
Gender	Male	1 (1.7)	4 (7.0)	15 (26.3)	11 (19.2)	6 (10.5)	1 (1.7)	1 (1.7)	0 (0)	0 (0)	1.663,6	0.948	
	Female	0 (0)	2 (3.5)	6 (10.5)	5 (3.5)	4 (7.0)	0 (0)	1 (1.7)	0 (0)	0 (0)			

## Discussion

Urinary stone disease represents a significant public health challenge, carrying the risk of progression to various urological conditions, including kidney failure in severe cases. The prevalence of urinary stone formation exhibits regional disparities: it ranges from 5% to 9% in Europe, 1% to 5% in Asia, 10% to 15% in the United States, and peaks at 20% to 25% in the Middle East [13]. Consistent with the study conducted by Jindal et al., our findings indicate a mean patient age of 38.5 years for males and 40.4 years for females, reinforcing male predominance in urinary stone cases within the eastern Indian population [14].

Large-scale investigations, including Knoll et al.'s cohort study, consistently highlight the predominance of calcium oxalate stones, closely followed by uric acid stones [15]. Ansari et al.'s study involving 1,050 renal stones from north Delhi further validates these findings, identifying calcium oxalate in an astounding 93.04% of cases [16]. Similar outcomes were established from studies by Tanthanuch et al. and Rahman et al., both emphasizing calcium oxalate as the most prevalent stone type [17-18].

Within the scope of our study, calcium oxalate emerged as the predominant stone type, followed by carbonate apatite stones. We also identified other stone components, including uric acid, struvite, calcium hydrogen phosphate, xanthine, and cystine. Detailed insight into stone composition significantly influences stone therapy selection. For instance, extracorporeal shock wave lithotripsy demonstrates enhanced efficacy in disintegrating calcium oxalate dihydrate and struvite stones, whereas cystine-rich stones require alternative approaches [19].

In certain instances, stone composition analysis can reveal metabolic disorders. Struvite stones correlate with chronic urinary tract infections [20]. When scrutinizing frequently encountered urinary stones, the diagnostic significance of their composition becomes imperative. These stones often have a combination of calcium oxalate and calcium phosphate [21]. Calcium phosphate stones are linked to renal tubular acidosis and hyperthyroidism, while uric acid stones are related to gout [22].

Urinary stone prevalence increases with age, peaking around 60 to 70 years and declining afterward [23, 24]. Notably, children aged between one to 10 years exhibit a distinct preference for ammonium hydrogen urate stones [25]. In our investigation, a discernible trend in urinary stone incidence emerged. It initiates an ascent in the 11 to 20 age group, peaks between 31 and 40 years, and declines in the 61 to 70 age group. Our study confirms that urinary stones predominantly affect the kidney, followed by the ureter and bladder, consistent with Chand et al.’s observation of 75.08% renal stones, 13.62% ureteric stones, and 1.74% bladder stones in their study population [26].

A study conducted by Tiyan et al. had observations similar to those of our study. They found that most stones analyzed had two components, followed by those with three components, while pure stones were the least common. Kidney stones were the most prevalent and were primarily treated with percutaneous nephrolithotomy. Calcium oxalate monohydrate and calcium oxalate dihydrate were frequently found in combination stones, while uric acid stones were predominant as pure components [27].

Dietary therapy offers a promising approach to mitigating urinary stone recurrence and improving patients' overall quality of life. Notably, in India, dietary patterns high in proteins and carbohydrates play a significant role in the prevalence of urinary stones. Coupled with inadequate fluid intake, these dietary choices contribute to stone formation. Raising awareness and implementing optimal dietary interventions can reduce hospitalization costs and enhance adherence to preventive measures. Thus, dietary factors and fluid intake are primary risk factors for urinary stone development [28]. Our study highlights the critical role of a comprehensive approach in the prevention of urinary stone formation. A significant reduction in stone recurrence was observed among participants who adhered to a dietary regimen low in animal protein coupled with adequate hydration and appropriate lifestyle modifications, similar to the findings reported by Risal et al. and Ferraro et al., suggesting that a multifaceted prevention strategy is essential for patients at high risk of recurrent stones. Implementing such an approach could improve patient outcomes and contribute to a reduction in healthcare costs [29,30].

This comprehensive analysis outlines the demographic and anatomical distribution of stone formation in the studied population and emphasizes the necessity for future research to investigate the relationship between diet and urinary stone formation to develop more effective preventive strategies.

## Conclusions

This study underscores that young males are particularly prone to developing urinary stone disease. Patients often present with classic symptoms such as colicky pain, nausea, and vomiting. The majority of urinary stones in patients are composed primarily of calcium oxalate. Importantly, the findings suggest that implementing a personalized dietary plan, ensuring adequate water intake, making appropriate lifestyle adjustments, and maintaining regular follow-up can significantly reduce the risk of stone recurrence and the need for major surgery in affected individuals. These preventive measures could play a crucial role in long-term management and improving patient outcomes.
